# Spatial spillover effects from agriculture drive deforestation in Mato Grosso, Brazil

**DOI:** 10.1038/s41598-021-00861-y

**Published:** 2021-11-08

**Authors:** Nikolas Kuschnig, Jesús Crespo Cuaresma, Tamás Krisztin, Stefan Giljum

**Affiliations:** 1 Vienna University of Economics and Business (WU), Welthandelsplatz 1, 1020 Vienna, Austria; 2 International Institute for Applied System Analysis (IIASA), Laxenburg, Austria; 3 Wittgenstein Centre for Demography and Global Human Capital (WIC), Vienna, Austria; 4 Austrian Institute of Economic Research (WIFO), Vienna, Austria

**Keywords:** Environmental economics, Environmental impact

## Abstract

Deforestation of the Amazon rainforest is a threat to global climate, biodiversity, and many other ecosystem services. In order to address this threat, an understanding of the drivers of deforestation processes is required. Spillover effects and factors that differ across locations and over time play important roles in these processes. They are largely disregarded in applied research and thus in the design of evidence-based policies. In this study, we model connectivity between regions and consider heterogeneous effects to gain more accurate quantitative insights into the inherent complexity of deforestation. We investigate the impacts of agriculture in Mato Grosso, Brazil, for the period 2006–2017 considering spatial spillovers and varying impacts over time and space. Spillovers between municipalities that emanate from croplands in the Amazon appear as the major driver of deforestation, with no direct effects from agriculture in recent years. This suggests a moderate success of the Soy Moratorium and Cattle Agreements, but highlights their inability to address indirect effects. We find that the neglect of the spatial dimension and the assumption of homogeneous impacts lead to distorted inference. Researchers need to be aware of the complex and dynamic processes behind deforestation, in order to facilitate effective policy design.

## Introduction

The Amazon rainforest is the world’s largest forest and provides a wide range of ecosystem services. It plays an important role in regional as well as global climate stabilisation^1–3^, is an unparalleled biodiversity hotspot^4,5^, and is essential to the livelihoods and health of people^6,7^. Deforestation is a threat to the continued provision of these services and thus a primary focus of environmental policy (see^8–10^). The rate of forest loss in the Amazon remains high (see e.g.^11^), making research on the determinants of deforestation in this part of the world particularly relevant. Environmental policy design requires an understanding of the nature of deforestation and linked processes, such as forest degradation, to support the role of forests in a variety of future challenges^12–14^.

Unveiling the drivers of deforestation has been on the research agenda for decades, with shifting focal points. Early studies of deforestation in the Brazilian Amazon emphasise the roles of infrastructure investment and governmental programmes, such as settlement policy and credit programmes^15,16^. These intensified the role of market forces as a determinant of the dynamics of land use change^17,18^. Large-scale cattle ranching and expanding croplands were identified as major drivers of deforestation (see e.g.^7,19,20^), motivating a number of policies to limit these adverse effects. The most prominent examples of such policies are zero-deforestation commitments, which seek to eliminate deforestation from supply chains (see^7,8,21^). Such command-and-control policies are the main form of intervention, as opposed to incentive-based policies^13^. However, their efficacy may be undermined by spillover effects, such as indirect land use change or leakage into other regions^22–24^.

**Figure 1:**
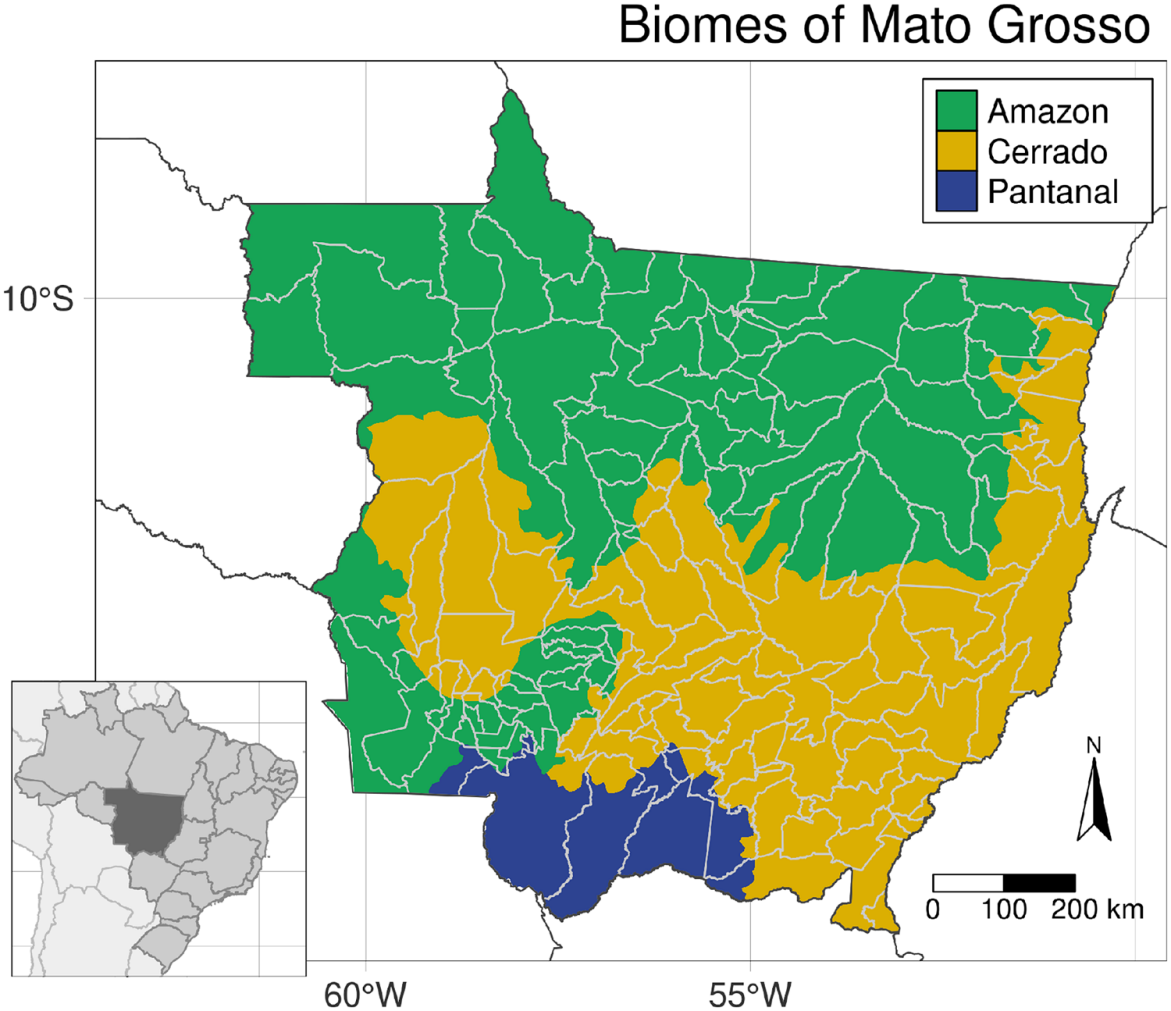
Mato Grosso lies at the intersection of the Amazon rainforest, the Cerrado tropical savannah, and the Pantanal wetlands (coloured). The frontier state is located in the centre-west of Brazil (see inset) and is subdivided in 141 municipalities (gray lines). Created using the **tmap** (version 3.3-2) R (version 4.1.1) package^25,26^.

Two particularly salient policies in this respect are the Soy Moratorium and the Cattle Agreements^7^. These voluntary zero-deforestation commitments seek to eliminate deforestation from the soy and cattle supply chains of participating companies. The Soy Moratorium bans soy from previously deforested areas in the Amazon biome (see Fig. 1) since its inception in 2006, with the Cattle Agreements following in 2009^8,21^. Both interventions were adapted and extended over time to achieve higher levels of compliance, applicability, and efficacy. A major challenge for their efficacy is indirect land use change. Croplands (and to an extent, pastures) displace other land use types that, in turn, replace forests, undermining the interventions^22,23^. Since interventions are limited to the Amazon, detrimental impacts may leak into other biomes, a phenomenon called leakage. Today, the neighbouring Cerrado biome is a deforestation hotspot^27^ and its native vegetation is being lost at high pace^28^. The estimated effects of the Soy Moratorium and Cattle Agreements are highly dependent on the particular time period and space under scrutiny, which is reflected in mixed results in the literature (e.g.^7,8,21–23,27,29^).

Recent empirical studies of deforestation benefit from the availability of fine-scale, spatially explicit data, but rarely investigate spatial spillover effects explicitly (see Busch and Ferretti-Gallon^30^, for a meta-analysis). Studies that do consider spillovers show a wide variety of indirect effects. Among them, Aguiar et al.^31^ and Hargrave and Kis-Katos^32^ take broad approaches, investigating deforestation using pooled data for the whole Amazon biome. Amin et al.^33^ assess the efficacy of protected areas in deterring deforestation. Gollnow et al.^34^ focus on finer scales, investigating indirect deforestation at property level in the state of Mato Grosso. A different approach is taken by de Sá et al.^35^, who examine spillover effects from sugarcane expansion in São Paulo state on forest conversion in the Amazon. Pronounced spatial dynamics arise as a consistent feature of such studies. Nonetheless, empirical studies that consider both spillover effects and heterogeneity of impacts in the context of the Soy Moratorium and Cattle Agreements are lacking.

In this study, we evaluate direct and indirect impacts of agriculture on deforestation in the Brazilian state of Mato Grosso. In our econometric framework, we explicitly consider spillovers both across municipalities and the whole state, and account for the temporal and spatial heterogeneity of effects stemming from different biomes, legislation, and evolving interventions. Our statistical analysis is carried out using a panel regression model with data at the municipal level that covers the period 2006–2017 on a yearly basis. We accommodate spatial spillovers in our modelling framework via spatial lags, and assess heterogeneity by allowing impacts to vary across biomes and time periods. Our main research questions are: (1) How does agriculture impact deforestation directly and indirectly? (2) Have these dynamics changed over time? and (3) What are the differences across biomes?

**Figure 2:**
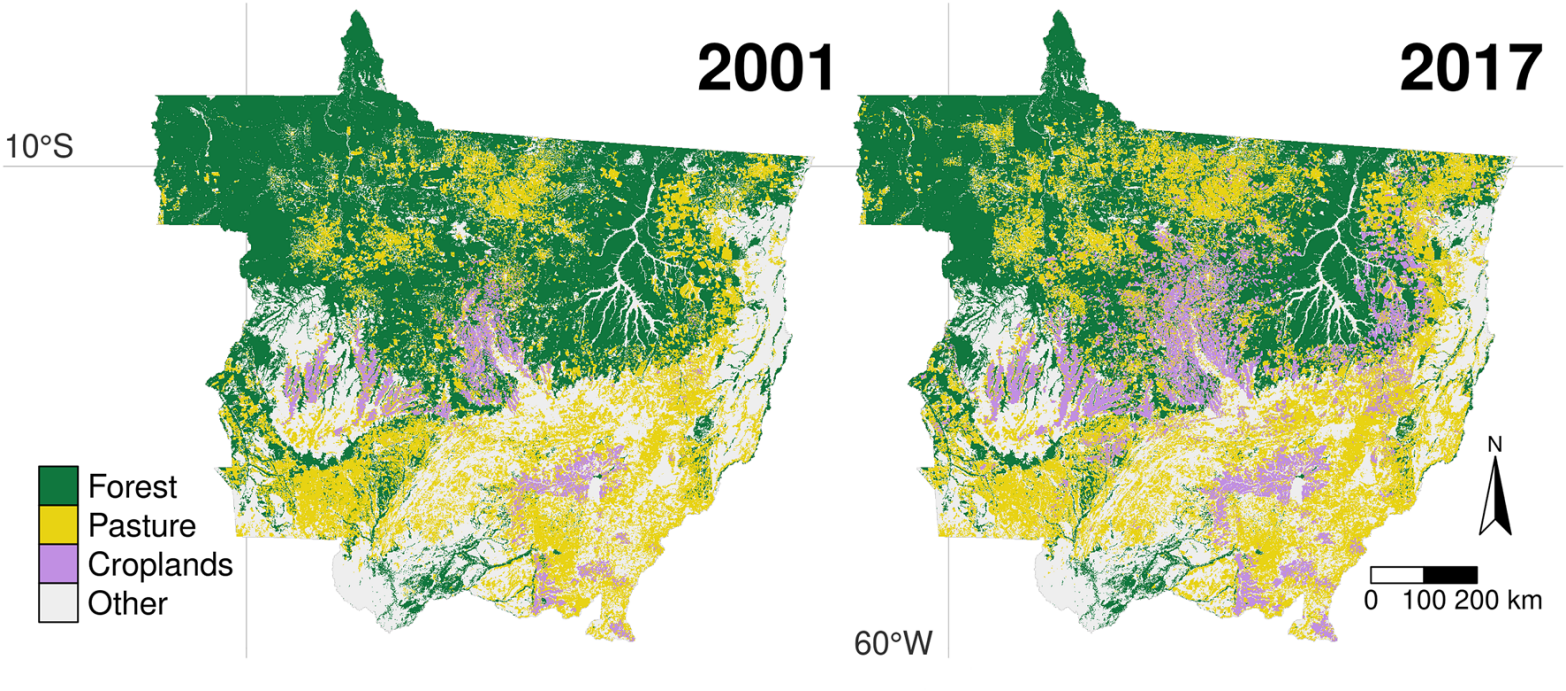
Land use cover in Mato Grosso in 2001 and 2017. The clustered expansion of croplands and their appearance in areas previously covered by forest or pasture is noticeable when comparing both years. The area devoted to pasture has also increased considerably, replacing vast amounts of forest. See Fig. 3 for a visualisation of transitions between land use cover. Data is derived from the maps by Câmara et al.^40^; created using the **tmap** (version 3.3-2) R (version 4.1.1) package^25,26^.

Mato Grosso lies at the intersection of the Amazon, Cerrado, and Pantanal biomes (see Fig. 1) and was an early deforestation hotspot (see e.g.^36,37^). After exceptional agricultural expansion and intensification^37,38^ it is the number one producer of soy and beef in Brazil today^39^. Mato Grosso is subject to exceptionally dynamic land use change—vast amounts of forest have made way to agricultural lands, which are predominantly used for the production of soy and beef, since the turn of the century (cf. Fig. 2). Mato Grosso is a particularly interesting region due to these rapid changes, its economic importance, varied geography, and the heterogeneous political climate in the context of the Soy Moratorium and Cattle Agreements. Insights from the region are important for understanding deforestation processes and informing policy design.

Our findings show considerable spatial spillovers in deforestation and substantiate agriculture as a driver of deforestation. The transmission of these impacts is varied, with cattle herds acting as a direct driver, and croplands as an indirect one, affecting neighbouring municipalities. Impacts from croplands are relatively stable throughout the studied period, while pressure from cattle disappears in more recent years. The overall dynamics are shaped in the Amazon biome and differ considerably from the Cerrado and Pantanal. There, deforestation is affected by direct impacts from cattle and pasture and incoming cropland spillovers from the Amazon. These results have strong implications for future studies on deforestation and environmental policy design. Empirical studies need to account for spatial spillovers in order to avoid distorted impact estimates. Heterogeneous effects need to be considered to obtain deeper and more precise insights on the efficacy of targeted policy interventions. Our results suggest that voluntary zero-deforestation commitments can complement national policy measures, but cannot replace them.

## Results

Indirect impacts from croplands are the main empirical driver of deforestation in Mato Grosso. Table 1 presents the overall effect estimates for pastures, croplands, cattle density, and soy yields. The significant (95% HPDI does not contain zero) agricultural factors behind deforestation are croplands, which are predominantly used for the cultivation of soy, and cattle herds. Impacts from croplands are indirect, with an estimate of − 8.083 hectare per square kilometre in forest change per cropland share (in percent of total area). Cattle have direct impacts, with an estimate of − 0.153 hectare per square kilometre of forest change per percent increase in cattle density. These impacts from cattle ranching are related to intensity, as measured by the number of cattle per pasture, instead of the mere presence of pasture, whose effect is statistically insignificant. In the case of soy production the share of croplands is the main driving factor of forest cover change, instead of soy yields. The spatial dynamics of deforestation processes are very pronounced in our sample. State-wide spillovers, direct impacts within a municipality, and indirect ones from neighbouring municipalities are important driving factors of forest cover changes.

**Table 1:** Regression of deforestation on agricultural drivers and control variables.

Forest change $\sim$	Direct			Indirect		
	Mean	(HPDI)		Mean	(HPDI)	
Croplands	0.941	(− 0.141	2.017)	− 8.083	(− 11.964	− 4.24)
Soy yields	0.055	(− 0.014	0.122)	0.131	(− 0.159	0.411)
Pasture	− 0.150	(− 0.886	0.553)	1.675	(− 1.116	4.583)
Cattle	− 0.153	(− 0.261	− 0.048)	0.190	(− 0.379	0.706)

We report the posterior mean and boundaries of credible intervals (the highest posterior density intervals (HPDI)) covering 95% of the posterior. Effects are divided into direct effects (within a municipality) and indirect effects (affecting other municipalities). Deforestation is measured as change of forest in hectare per total area in km^2^, croplands and pasture in percent of area, cattle as logged cattle density per pasture, and soy yields in Brazilian Real per harvested area. More information on the model, data, and extended results are provided in the methods section and supplementary material.

The magnitude of agricultural impacts is considerable. As an illustration, consider a scenario where the impacts of croplands or cattle are reduced by ten percent in 2017. In 2017 we observe an average forest cover loss of 0.150 hectare per km^2^, for a total of 1402 km^2^. Our model predicts an average of 0.174 hectare per km^2^, corresponding to total deforestation of 1514 km^2^. Reducing indirect impacts of croplands by ten percent is equivalent to avoiding 1122 km^2^ of deforestation (i.e. the prediction for deforested area is reduced to 392 km^2^). For direct impacts of cattle, the prediction is 509 km^2^ of avoided deforestation (1005 km^2^). If we consider total impacts instead of indirect or direct ones, we find 679 km^2^ of avoided deforestation for croplands and 37 km^2^ of additional deforestation for cattle. These magnitudes differ considerably from models where spatial spillover effects are not considered. Using a standard linear regression model without connectivity, the cropland scenario yields 165 km^2^ of avoided deforestation, and the cattle scenario 125 km^2^. The omission of spatial dynamics thus results in a severe underestimation of the deforestation impacts of croplands relative to cattle and of agriculture in total.

**Table 2:** Regressions with heterogeneous effects across biomes and over time periods.

Specification A	Forest change $\sim$	Direct			Indirect		
		Mean	(HPDI)		Mean	(HPDI)	
Amazon	Croplands	1.967	(0.604	3.365)	− 8.443	(− 13.260	− 3.754)
	Soy yields	0.069	(− 0.006	0.142)	0.148	(− 0.151	0.449)
	Pasture	0.885	(− 0.044	1.876)	3.123	(− 0.887	6.882)
	Cattle	− 0.243	(− 0.393	− 0.093)	0.063	(− 0.734	0.871)
Other	Croplands	0.121	(− 1.305	1.608)	− 3.831	(− 10.811	2.957)
	Soy yields	0.043	(− 0.031	0.116)	0.084	(− 0.192	0.376)
	Pasture	− 1.111	(− 2.024	− 0.198)	− 1.130	(− 4.920	2.144)
	Cattle	− 0.089	(− 0.233	0.070)	0.115	(− 0.658	0.866)

Specification B	Forest change $\sim$	Mean	(HPDI)		Mean	(HPDI)	
2006–2011	Croplands	− 0.016	(− 1.230	1.240)	− 9.857	(− 14.753	− 4.876)
	Soy yields	0.198	(0.048	0.339)	0.573	(− 0.044	1.228)
	Pasture	− 0.281	(− 0.994	0.466)	3.089	(0.285	6.096)
	Cattle	− 0.197	(− 0.306	− 0.076)	0.425	(− 0.154	0.981)
2012–2017	Croplands	0.638	(− 0.529	1.772)	− 8.851	(− 13.825	− 4.322)
	Soy yields	0.016	(− 0.057	0.088)	0.031	(− 0.275	0.330)
	Pasture	0.057	(− 0.644	0.821)	3.005	(0.098	6.120)
	Cattle	− 0.075	(− 0.190	0.046)	0.369	(− 0.201	0.935)

Specification A on the top allows effects for the Amazon biome to differ from the rest of the state. Specification B on the bottom allows different effects for the 2006–2011 and 2012–2017 periods. See Table 1 for further information.

Heterogeneity of deforestation impacts plays an important role in explaining the dynamics of forest cover loss in the region. We find considerable differences for different time periods and for the Amazon biome. Table 2 presents effect estimates that allow for variation of effects across biomes and over time periods.

The results presented at the top of Table 2 summarise the estimated impacts using a specification that allows for biome-specific effects. The Amazon biome largely mirrors the pooled results for the whole of Mato Grosso. Croplands appear as the most important driver, with considerable indirect effects and, as a result, total effects. The effect estimate of − 8.443 is comparable to that of the pooled model. The effects of cattle are also comparable: direct effects are a significant driver, but indirect effects are more uncertain. The Amazon-specific impacts of pasture are positive, but remain insignificant. This contrasts with the evidence for the other biomes, where pasture emerges as a significant direct driver of deforestation, although total effects remain ambiguous. By contrast, the effects of croplands, direct and indirect, are insignificant outside of the Amazon biome.

The bottom of Table 2 holds the results with period-specific estimates for the periods 2006–2011 and 2012–2017. The earlier period corresponds to the years when the Soy Moratorium and Cattle Agreements came into effect. The period is characterised by higher deforestation rates, at an average of 4085 km^2^ of deforested area per year. The later period has both zero-deforestation commitments in full effect and displays lower deforestation rates, with an average of 1748 km^2^ per year. In both periods, cropland density appears as a driver of deforestation with considerable indirect and total effects. However, the magnitude of effects drops off in the later period. For 2006–2011, high soy yields significantly deter direct deforestation. In the case of cattle, we only find significant direct effects for the earlier period and no significant total effects. With estimates of 3.089 and 3.005, pasture acts as a significant indirect deterrent to deforestation in both periods. The link between animal husbandry and deforestation is ambiguous in the earlier period and from 2012 until 2017 no significant effects are found.

**Figure 3:**
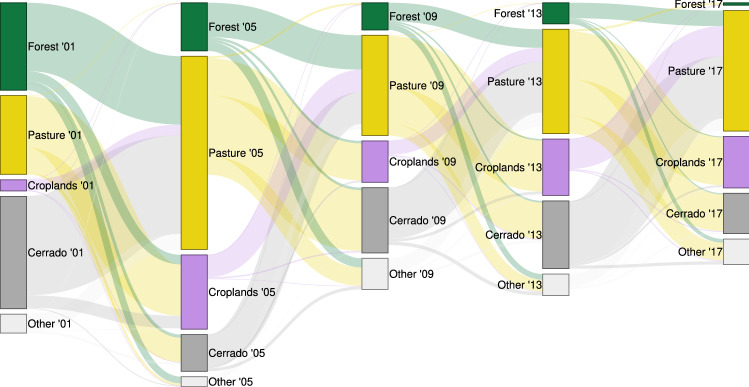
Direct land use change over selected years from 2001 to 2017. The height of flows represents the amount of yearly incoming/outgoing land use cover, the height of individual bars the maximum thereof. An interactive version of this figure with exact measurements of flows as tool-tip is provided as supplementary material. Also see Fig. 2 for a visualisation of land use cover in 2001 and 2017.

The importance of spillover effects is best understood when contrasted to direct land use changes as depicted in Fig. 3. Early on, over the period 2001–2005, a large amount of forest is converted directly into pasture (26,304 km^2^) and croplands (3610 km^2^). In subsequent years, the pace of forest conversion slows down. In particular, the conversion of forest to croplands largely ceases while indirect impacts persist. Croplands are a consistent net-receiver of land use change over the investigated period. This contrasts with forests, which consistently suffer from a large net-loss amidst large outflows and minuscule inflows. Among the outflows we find conversion into pasture, which is itself converted into croplands, as the most significant determinant. We identify a major pathway behind these deforestation processes in the form of spatial spillover effects.

## Discussion

Our analysis reveals a spatially complex and heterogeneous picture of deforestation dynamics in Mato Grosso. Agriculture remains pervasive in shaping the region’s landscape, but pathways have changed over the past 20 years. Spillover effects from agriculture, long theorised and confirmed statistically by 23, are the main empirical driver of continued deforestation today. Conversion of forest to pastures is an important factor, but is being driven indirectly by croplands. Within the group of agricultural commodities, soy emerges as the dominant driver of deforestation. Direct impacts from cattle only emerge in the 2006–2011 period, when the Cattle Agreements were not yet in full effect. In the 2012–2017 period, we observe slightly lower impacts of croplands and agriculture overall. These results are mainly driven by dynamics in the Amazon biome. Effects in the Cerrado and Pantanal biomes differ considerably, with pasture and cattle as the sole significant deforestation drivers. Spatial connectivity and heterogeneity in effects have strong implications for the construction of empirical models of deforestation, as well as for the design of environmental policy responses.

Spillover effects and heterogeneity play important roles in deforestation processes, but are not systematically assessed in empirical studies (see^30^). When considered, spillover effects—both between municipalities and across the whole state—turn out to be an important piece of the deforestation puzzle, as evidenced by some previous works (e.g.^23,31–33^) and by our study for the case of Mato Grosso. We find that disregarding spillovers leads to an underestimation of the impacts of agriculture and a relative underestimation of the importance of croplands. Focusing on the state of Mato Grosso, heterogeneity of effects over time and space is pronounced. Most previous studies operate on larger spatial scales that arguably imply more heterogeneity, but largely ignore the issue in their modelling frameworks (cf.^30^). By wrongly assuming homogeneity of effects, a range of important impacts may remain hidden behind average results. The lack of a consistent and effective treatment of spatial connectivity and heterogeneity casts some doubts on many of the empirical results obtained hitherto. Our statistical model considers these elements and yields important additional insights into the nature of deforestation processes and their drivers. Indirect deforestation pressure continues to be rampant in Mato Grosso, with direct pressure waning. Spillover effects emanating from croplands in the Amazon are the main driver of deforestation in the region.

The Cattle Agreements and particularly the Soy Moratorium are generally understood to have been successful in alleviating direct deforestation pressure^7,8,21,29^. However, there are serious doubts regarding the overall success due to spillover effects^22–24,41^. Our study corroborates both stories. In later years, deforestation pressure from agriculture is exclusively indirect, but deforestation and other detrimental impacts of land use change remain problematic. More comprehensive measures to curb deforestation are needed urgently. There is some debate on voluntary zero deforestation commitments (e.g.^28,41^) and the role of certain commodities and trade (e.g.^27,42^) in deforestation. Measures targeted at specific drivers may serve as short-term remedies, but lasting solutions require more comprehensive governmental action. As a common good, the Amazon rainforest will continue to suffer from exploitation as long as there is value to be extracted^43^. If protective measures are overly focused, new deforestation drivers may arise imminently. Alternative policies that tackle the issue at its root exist, e.g. land use or carbon taxes, and could effectively and efficiently limit deforestation^13^.

Future research will have to shed light on a number of open issues around deforestation. In this study, we concentrated on Mato Grosso, but larger scale analyses may offer additional insights. These could ensure the external validity of the results presented, but must not come at the loss of internal validity, e.g. due to omitting heterogeneity or spillover effects. An important issue to address is the heterogeneity of spillover effects, which could occur, e.g., on regional, national, or even international scales. Further attention to natural vegetation other than forests is also needed. The Cerrado’s native vegetation is being converted at high pace^28^ and Song et al.^38^ find that the cultivation of soy in the Pantanal wetlands is rapidly expanding. These processes evolve in parallel to Amazon deforestation and additional insights may help addressing adverse effects and developing suitable countermeasures. Our study investigates agricultural impacts related to soy and cattle, the major commodities whose impacts are being addressed in zero deforestation commitments. However, it is important to understand deforestation drivers in a broad context, since deforestation-inducing commodities are manifestations of a market that places little value on forests. Researchers and policymakers alike need to be aware of this fact and the spillovers, leakages, and other complexities in deforestation that come with it.

## Methods

The data used for the study cover the 141 municipalities of Mato Grosso (Brazil) over the course of twelve years ($N = 1692$). We investigate the impacts of agriculture on deforestation using a spatial panel model. The panel structure and our dataset allow us to account for relevant observed and unobserved factors affecting deforestation in the period considered. The spatial specification captures indirect factors in deforestation processes and is therefore able to model impacts that may spill over municipal borders.

### Econometric model

The econometric model is of the form

1 $$\begin{aligned} {\mathbf {y}}_t = \rho \mathbf {Wy}_t + {\mathbf {X}}_{t-s} \mathbf {\beta } + \mathbf {WX}_{t-s} \mathbf {\theta } + \mathbf {\mu } + \mathbf {\psi }_t + \mathbf {\varepsilon }_t, \end{aligned}$$

where ${\mathbf {y}}_t$ is an $N \times 1$ vector with the yearly change in forest area in hectare per km^2^ at time *t*, ${\mathbf {X}}_t$ is an $N \times K$ design matrix with variables of interest and controls, and $\mathbf {\varepsilon }_t$ is an error term that is assumed to follow a multivariate Gaussian distribution with zero mean and a diagonal variance-covariance matrix ${\mathbf {I}} \sigma ^2$. The vectors $\mathbf {\mu }$ and $\mathbf {\psi }_t$ contain region-specific and year fixed effects, capturing space and time-invariant unobserved effects, respectively (see^44^). This model differs from standard models in that it explicitly assesses the potential existence of geographical spillovers by the use of a spatial connectivity matrix ${\mathbf {W}}$, which is used to impose a neighbourhood structure across observations (see^45^). The exact form of ${\mathbf {W}}$ is presented and discussed in the supplementary material. We include a spatially autoregressive lag $\mathbf {Wy}$ and a spatial lag of explanatory variables $\mathbf {WX}$, with respective parameters $\rho$ and $\theta$.

The model in Eq. (1) is termed the ‘spatial Durbin model’ (SDM) and nests other widely applied spatial model specifications, as well as the classical linear model^46^. In this framework, spillover effects may arise globally, i.e. across all observations, and locally, i.e. from neighbour to neighbour. The spatial autoregressive term induces a non-linear spatial filter. Partial effects are hence not given directly by the coefficients in Eq. (1). We report the summary statistics of average effects, which can be separated into direct (within a region) and indirect ones (in other regions) (see^46^). Estimation is performed using Bayesian methods. We report 95 percent Bayesian credible intervals (highest posterior density intervals) as measure of uncertainty. Further information on the estimation, including the priors used and full posterior densities of the estimates, is provided in the supplementary material.

### Variables

The design matrix includes variables that allow us to isolate the impact of agriculture on deforestation, while avoiding confounding effects from other factors that affect deforestation. These control variables were chosen based on the meta-analysis of deforestation drivers by Busch and Ferretti-Gallon^30^, previous studies of the region (e.g.^23,31,34^), and our particular econometric approach. The factors of interest—effects of agriculture are represented by four different variables. These are the areas devoted to (1) pasture and (2) croplands as percentage of total area, (3) the logged headcount of thousand cattle per pasture, and (4) soy-yields in Brazilian Real (BRL) per harvested hectare. Agriculture in Mato Grosso is dominated by cattle and soy; other types of livestock, crops like sugarcane, and forestry only play marginal roles in the region (c.f.^47^).

Control variables can be summarised along three broad categories (loosely following^30^): socioeconomic variables, infrastructure, and biophysical characteristics. From the first category, we consider the roles of population density and municipal gross domestic product per capita. We find little relevance for either, but keep population density to prevent confounding from effects driven by anthropogenic activity. Protected areas, which may also impact deforestation, are essentially constant over the studied time frame (calculations based on^48^ and hence captured in the fixed effects of our panel specification. We assume that other important policy measures generally occur at the national or state level and are hence accounted for given the granularity of our data and the use of fixed effects. We differentiate between large scale infrastructure, which opens up frontier regions and is generally considered to be exogenous^30^, and small scale infrastructure. The former are captured via regional fixed effects, while we omit the latter as a mediator between agriculture and deforestation. Biophysical characteristics, such as elevation, slope, and soil, are constant over the investigated period and hence absorbed by the fixed effects in the setting of our panel data model. This is only partially true for the effects of climate. Droughts, in particular, show variation over time and space and may affect deforestation^49^. To account for such effects, we construct an indicator for the incidence of particularly dry periods.

We investigate heterogeneous impacts of agriculture across biomes and over different time periods by interacting variables of interest with indicators for biomes and periods. First, the Amazon biome is likely to have distinct deforestation dynamics from the Cerrado and Pantanal, e.g. due to differences in forest code (see e.g.^7^). Different impacts of agriculture may be the consequence. This could result in increased uncertainty of estimates or hide important insights behind an inadequately averaged effect. We also consider heterogeneity of the Pantanal, but find very similar dynamics to the Cerrado and thus pool the two biomes. Second, deforestation dynamics may change over time. Particularly, the implementation of zero-deforestation commitments has evolved considerably in the years considered in our analysis. Important dates are the inception of the Soy Moratorium in 2006 and the Cattle Agreements in 2009, although adaptations and updates mean that they came into full effect later on. Gradual changes may be a factor behind the findings of Arima et al.^23^, who investigate the efficacy of the Soy Moratorium from 2003 until 2008, and le Polain de Waroux et al.^24^ and Alix-Garcia and Gibbs^22^, who look at the periods 2001–2013 and 2007–2015. These studies find little evidence for decreasing adverse impacts from environmental regulations. However, if interventions only come into effect gradually, their effects may be misjudged due to temporal heterogeneity.

### Data

Our dataset covers 141 municipalities of Mato Grosso on a yearly basis from 2006 until 2017. It is derived from three major sources: IBGE^47^ with various municipal statistics and maps, and the Standardised Precipitation-Evapotranspiration Index (SPEI)^50^, a multi-scalar drought index, and Câmara et al.^40^ with remotely sensed land cover change maps.

The IBGE^47^ data is commonly used in studies on deforestation and land use change (e.g.^31^). The datasets used are the *Censo Demográfico*, *Estimativas de População*, *Produto Interno Bruto dos Municípios*, *Censo Agropecuário*, *Produção Agrícola Municipal*, and *Pesquisa da Pecuária Municipal*. The SPEI is used to construct indicators for particularly wet or dry months and is described in detail by Vicente-Serrano et al.^51^. For our analysis, we aggregate individual pixels from the Câmara et al.^40^ dataset to the municipal level and derive land use cover variables (forest, pasture, and croplands). A visual comparison of the variables obtained at the start and end of the dataset is presented in Fig. 2.

The remotely sensed land cover change maps of Câmara et al.^40^ are available at a 250m resolution from 2001 to 2017 and use the MODIS sensor as source material for time consistency. Simoes et al.^52^ discuss the dataset in detail. The maps are generated with a support vector machine model for classification and are post-processed using Bayesian smoothing, land cover masks, and land use change calculus. The overall degree of accuracy is 96%, with user (producer) accuracy of 99% (98%) for forest, and 97% (98%) for pasture. We find that our aggregated cropland variable (the dataset distinguishes seven types of cropland) correlate strongly with official statistics at the municipal level^47^, as documented by Simoes et al.^52^. They also cross-check the dataset against state-of-the-art remote sensing data^53,54^ as well as governmental surveys and find them to be consistent and reliable.

In our analysis, we equate deforestation and the change of forest cover. Other types of forest loss are included, but play a minor role in practice. We find that 92% of non-minor forest loss in the region is due to deforestation or shifting agriculture, as defined by Curtis et al.^55^. Negative loss may occur due to the independent generation of yearly maps, but we observe few instances of gain in forest cover, and of small magnitude. Further information on aggregation, transformations used, exact variable descriptions, and summary statistics are provided in the supplementary material.

## Supplementary Information

Supplementary Information 1.

Supplementary Information 2.

Supplementary Information 3.

Supplementary Information 4.
