# Supplementary material for: Spatial spillover effects from agriculture drive deforestation in Mato Grosso, Brazil
Source: Sci Rep. 2021 Nov 8;11:21804. doi: 10.1038/s41598-021-00861-y (PMC8575964; doi:10.1038/s41598-021-00861-y)
Supplement: Supplementary file 3 — Supplementary Information 3. [file 41598_2021_861_MOESM3_ESM.html]

Land use change
