# Supplementary material for: Spatial spillover effects from agriculture drive deforestation in Mato Grosso, Brazil
Source: Sci Rep. 2021 Nov 8;11:21804. doi: 10.1038/s41598-021-00861-y (PMC8575964; doi:10.1038/s41598-021-00861-y)
Supplement: Supplementary file 4 — Supplementary Information 4. [file 41598_2021_861_MOESM4_ESM.pdf]

Supplementary Information
Spatial spillover effects from agriculture drive deforestation in Mato Grosso, Brazil

Nikolas Kuschnig*, Jesús Crespo Cuaresma,
Tamás Krisztin, and Stefan Giljum

* Correspondence to nikolas.kuschnig@wu.ac.at, Welthandelsplatz 1, 1020 Vienna, Austria.

Data

The raster data was aggregated to the municipal level using the R [5] packages **sf** [4] and **raster** [2]. All maps were visualised using **tmap** [7]. Soy yields derived from IBGE [3] data were imputed for observations without soy production. For regions with few missing values temporal interpolation was used; for regions without soy production spatial interpolation was used. The Standardised Precipitation-Evapotranspiration Index (SPEI) was obtained from `https://spei.csic.es/` and aggregated to the municipal level. The analysis uses the three month scale of the SPEI, which is related to soil water content, headwater discharges, reservoir storage, and river discharges. The constructed indicator variables for particularly wet or dry months are defined as SPEI values below or above the critical values of -2 and 2 in a municipality in a year. Land use cover variables were derived from the maps of Câmara et al. [1] directly (forest, pasture) and as a union of all crop classifications (croplands). Crop classifications are soy, alone and double-cropped with corn, cotton, millet, or sunflower, and cotton as well as sugarcane. Simoes et al. [6] find maps to be accurate at the 250m level. We form a single crop classification and aggregate pixels to the municipal level, making the information less granular and even more accurate on the level of the analysis. Variable descriptions, summaries, and a visualisation of forest change can be found in Table S2, Table S4, and Figure S2.

Estimation

The connectivity matrix $\mathbf{W}$ is an $N \times N$ matrix with entries $w_{ij} > 0$ for neighbouring observations i and j where $i \neq j$ and $w_{ij} = 0$ otherwise. Matrices were constructed using Queen contiguity and transformed to be row-stochastic. Results are robust to adjusting

the connectivity matrix to various k nearest neighbour specifications. The probability model uses a Normal Inverse-Gamma prior for the nested linear model and a Beta prior, transformed to be on the interval $(-1, 1)$, for the spatial autoregressive parameter ρ. The setup reads as follows:

$$\begin{aligned}\vartheta &\sim \mathsf{Normal}(\mathbf{b}_0, \mathbf{B}_0),\\ \sigma^2 &\sim \mathsf{Gamma}^{-1}(c_0, d_0),\\ \rho &\sim \mathsf{Beta}(a_0, a_0).\end{aligned}$$

where ϑ collects the coefficients of the nested linear panel model. Prior parameters are chosen to be uninformative for the setting, i.e. $\mathbf{b}_0 = \mathbf{0}$, $\mathbf{B}_0 = diag(10^6)$, $c_0 = 2$, $d_0 = 1$, $a_0 = 1.01$. Results are robust to adjusting the prior parameters. The posteriors are not analytically tractable and estimation was performed using Markov chain Monte Carlo (MCMC) methods with a Griddy-Gibbs step. Chains used 25,000 draws, of which 5,000 were discarded as burn-in. Proper convergence was assessed using trace and density plots as well as standard convergence diagnostics.

Results

The full posterior densities for parameters of the SDM specification in Table 1 are visualised in Figure S3. Parameters and measures of model fit for this and various other models are also provided in Table S3. The necessity for a spatial autoregressive term was cross-checked using the test statistics of Moran's I and Geary's C (see Table S3). Results were checked for robustness against varying specifications, connectivity matrices, and prior setups. Extended results using different connectivity matrices, variables, and functional forms as well as robustness checks are available from the authors upon request.

Reproducibility

Code and data to produce the results of this paper are available as supplementary material. The full codes to reproduce the whole analysis are available in a version controlled repository at https://github.com/nk027/deforestation_mt.

Supplementary Material

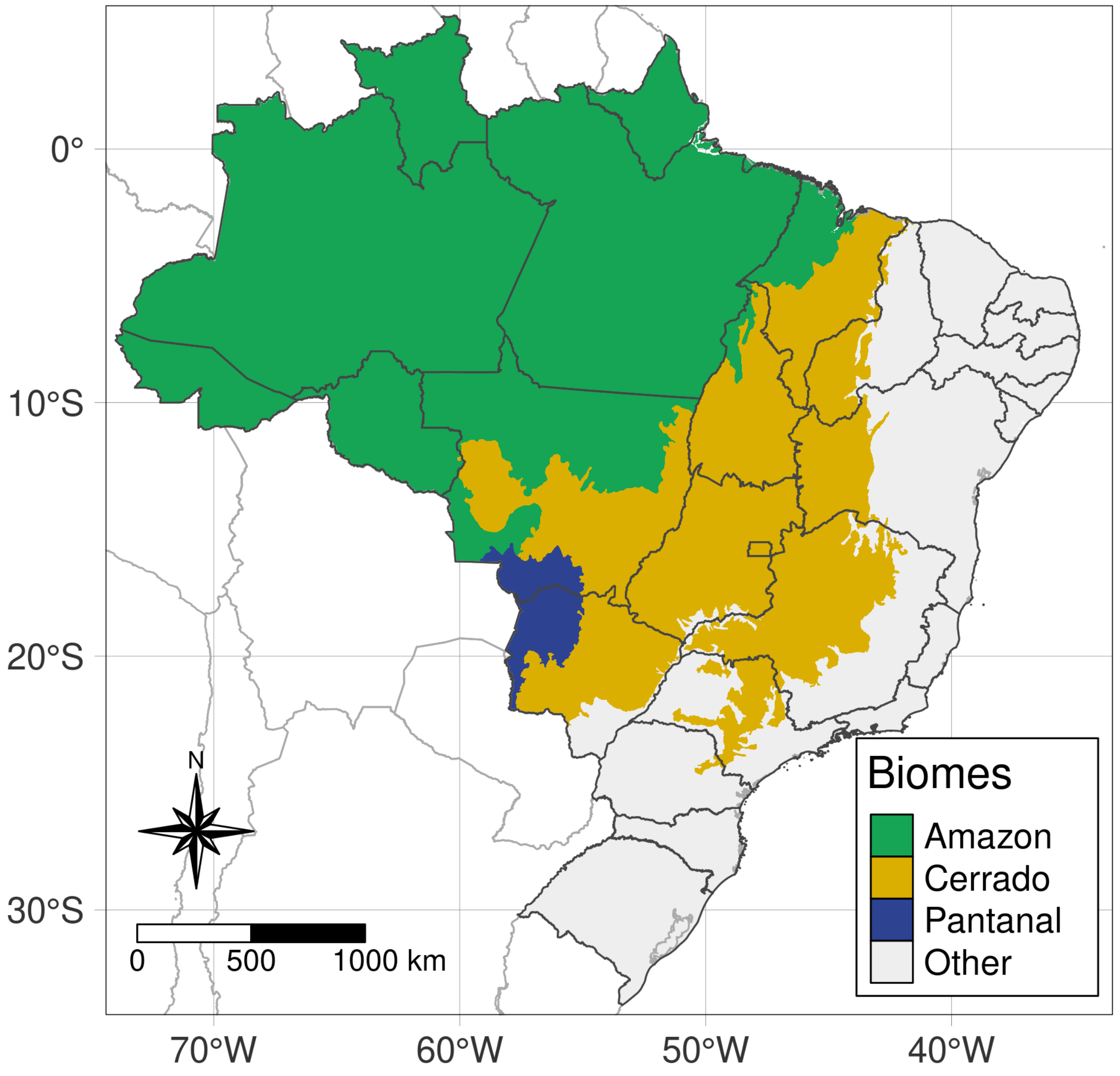

Figure S1: Brazil and its states with the Amazon, Cerrado, and Pantanal biomes highlighted. Mato Grosso lies in a unique position, at the intersection of the three biomes. Created using the **tmap** (version 3.3-2) R (version 4.1.1) package [5, 7].

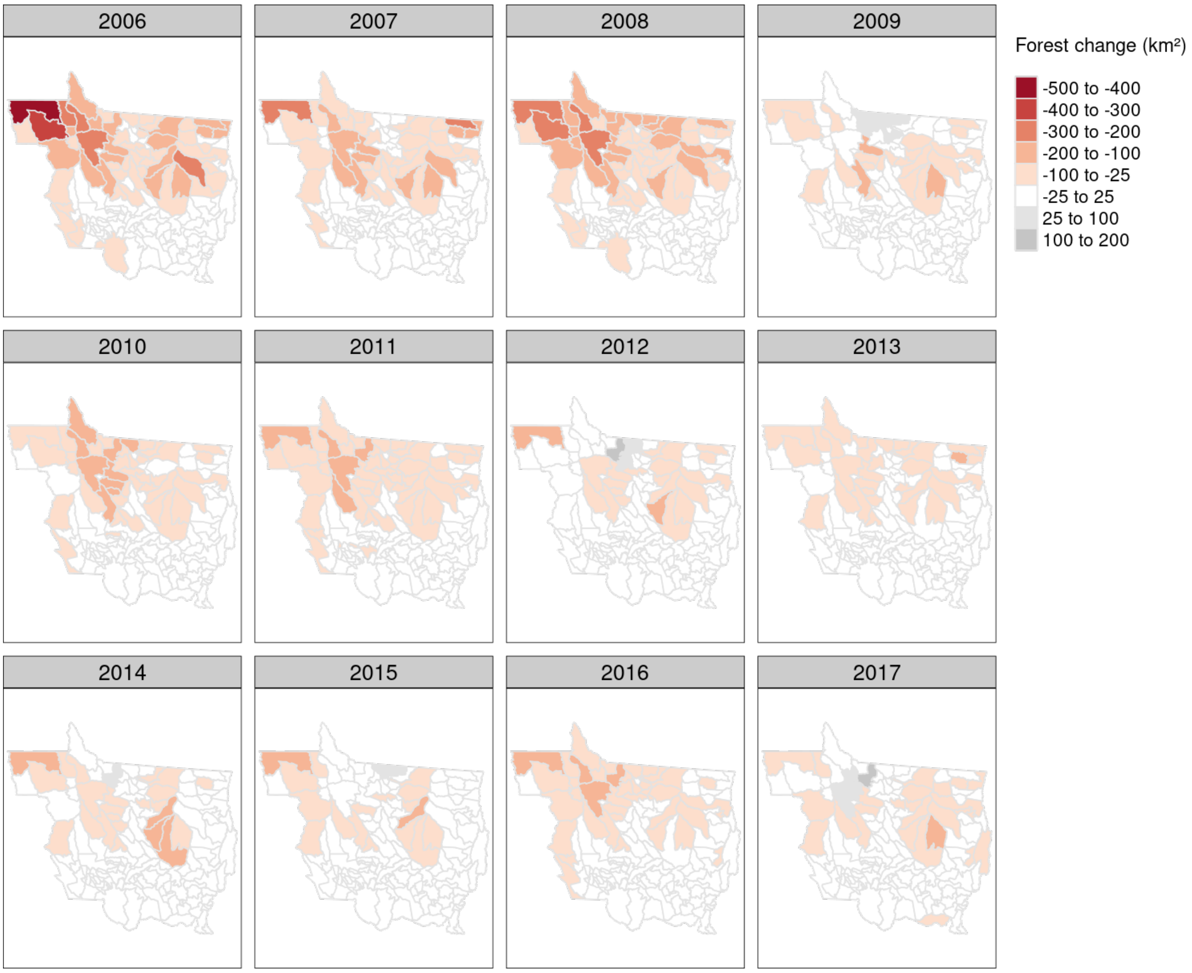

Figure S2: Yearly forest change in square kilometres over the investigated time period. Created using the **tmap** (version 3.3-2) R (version 4.1.1) package [5, 7].

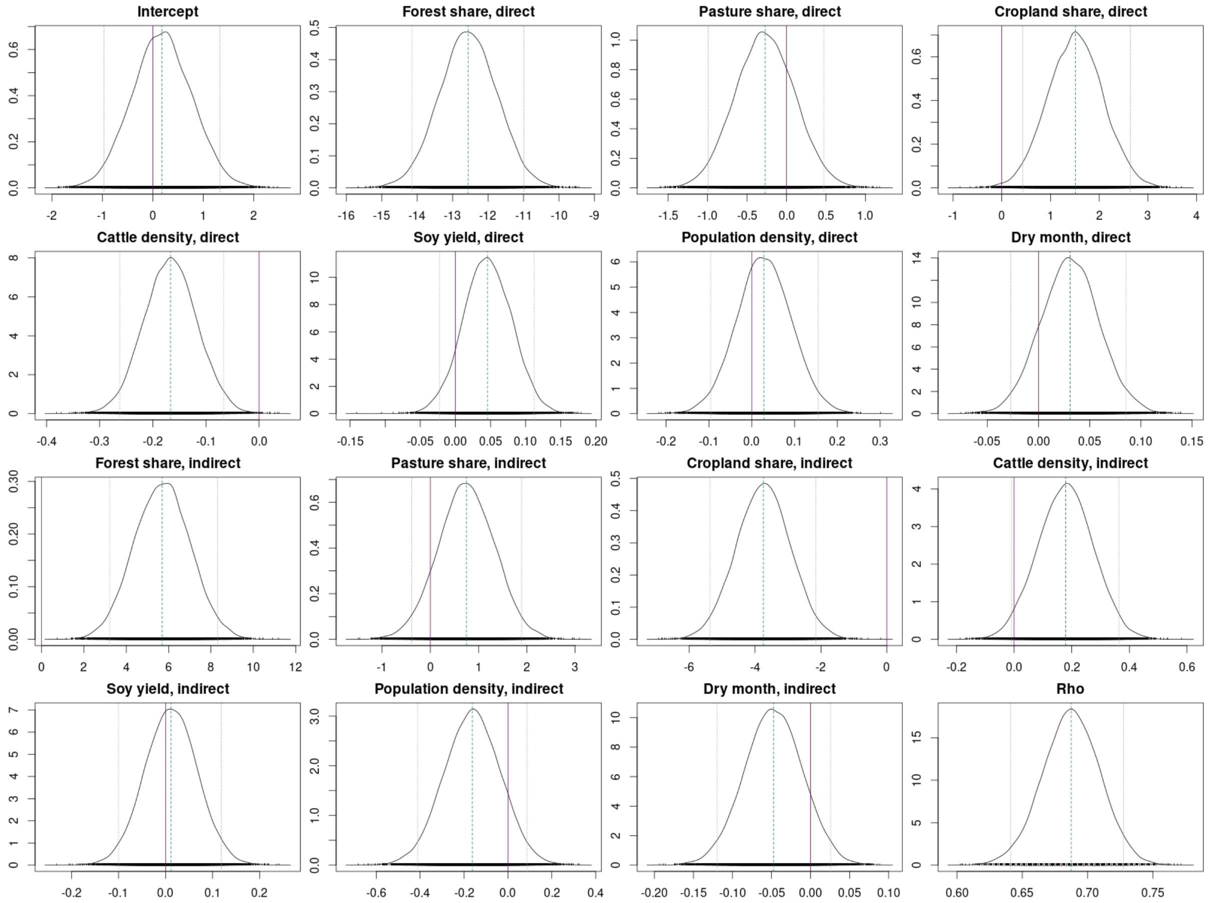

Figure S3: Posterior densities of all parameters of the main specification. Vertical lines indicate the posterior mean (teal, dashed), 95% HPDI (gray, dotted), and the origin (red).

Forest ~	SDM			SAR			SLX			CLM		
	Mean	(HPDI)		Mean	(HPDI)		Mean	(HPDI)		Mean	(HPDI)	
Intercept	0.182	(-0.968	1.327)	0.890	(0.260	1.512)	0.881	(-0.632	2.363)	0.660	(-0.163	1.441)
**Direct**												
Forest share	-12.559	(-14.148	-10.990)	-10.105	(-11.394	-8.791)	-13.183	(-15.234	-11.137)	-16.050	(-17.685	-14.479)
Pasture share	-0.271	(-0.991	0.472)	-0.556	(-1.147	0.000)	-0.016	(-0.981	0.916)	-0.301	(-1.032	0.435)
Cropland share	1.514	(0.428	2.639)	-0.899	(-1.717	-0.071)	1.149	(-0.241	2.598)	-1.890	(-2.956	-0.863)
Cattle density	-0.167	(-0.262	-0.066)	-0.119	(-0.215	-0.026)	-0.168	(-0.287	-0.037)	-0.131	(-0.257	-0.013)
Soy yield	0.046	(-0.023	0.112)	0.073	(0.016	0.131)	0.055	(-0.032	0.143)	0.098	(0.024	0.176)
Pop. density	0.029	(-0.096	0.156)	-0.035	(-0.158	0.091)	0.034	(-0.127	0.192)	0.000	(-0.150	0.171)
Dry month	0.031	(-0.027	0.086)	-0.011	(-0.045	0.020)	0.014	(-0.058	0.087)	-0.007	(-0.049	0.035)
**Indirect**												
Forest share	5.698	(3.215	8.318)				-7.665	(-10.796	-4.541)			
Pasture share	0.749	(-0.385	1.895)				0.112	(-1.372	1.579)			
Cropland share	-3.745	(-5.366	-2.159)				-6.737	(-8.799	-4.682)			
Cattle density	0.179	(-0.010	0.363)				0.099	(-0.147	0.344)			
Soy yield	0.012	(-0.1	0.118)				0.083	(-0.058	0.226)			
Pop. density	-0.162	(-0.412	0.088)				-0.184	(-0.528	0.121)			
Dry month	-0.047	(-0.120	0.026)				-0.039	(-0.131	0.058)			
ρ	0.687	0.641	0.728	0.648	0.607	0.689						
$RMSE$	0.259	0.255	0.263	0.267	0.264	0.271	0.335	0.331	0.339	0.343	0.340	0.347
BIC	1484			1523			2169			2205		

Table S1: Main regression table for different models — the spatial Durbin model (SDM), spatial autoregressive model (SAR), spatially lagged explanatory model (SLX), and the classical linear model (CLM). Shown are the posterior means of all regression coefficients, as well as highest posterior density intervals covering 95 percent of the posterior.

Variable	Description	Source
Forest change	Change of forest (ha) per area (km^2).	[1]
Forest share	Share of forest (km^2) per area (km^2).	[1]
Pasture share	Share of Pasture (km^2) per area (km^2).	[1]
Cropland share	Share of Croplands (km^2) per area (km^2).	[1]
Population density	Population per area (logged capita per km^2).	[3]
Cattle density	Cattle per pasture (logged head in thousands per km^2).	[3]
Soy yield	Yield per harvested area of soy (thousand 2010 BRL per ha).	[3]
Dry month	SPEI indicating at least one dry month (binary).	[8]

Table S2: Variables used in the analysis, descriptions thereof, and their sources.

Connectivity	Moran's I	p-value	Geary's C	p-value
Queen-contiguity	0.5852	$< 2.2e - 16$	0.3693	$< 2.2e - 16$
5-nearest-neighbours	0.6273	$< 2.2e - 16$	0.4315	$< 2.2e - 16$
7-nearest-neighbours	0.5871	$< 2.2e - 16$	0.4447	$< 2.2e - 16$

Table S3: Results of Moran's and Geary's test for spatial autocorrelation in the dependent variable. Both tests reject the null of no spatial autocorrelation for any of the considered connectivity matrices. The k-nearest-neighbour matrices were transformed to $1/2(\mathbf{W} + \mathbf{W}')$ for the calculation of Geary's C.

Variable	Min	1st Quintile	Mean	Median	4th Quintile	Max
Forest change	-4.225	-0.525	-0.293	-0.072	0.000	1.792
Forest share	0.000	0.018	0.251	0.177	0.488	0.940
Pasture share	0.028	0.184	0.382	0.366	0.544	0.968
Cropland share	0.000	0.001	0.091	0.023	0.169	0.648
Population density	-1.388	-0.006	0.897	0.801	1.702	5.553
Cattle density	2.428	4.180	4.596	4.628	5.077	5.584
Soy yield	0.112	1.129	1.890	1.872	2.587	5.098
Dry month	0.000	0.000	0.426	0.000	1.000	1.000

Table S4: Summary statistics of the variables used.

References

[1] Câmara, G., Picoli, M., Maciel, A., Simoes, R., Santos, L., Andrade, P. R., Ferreira, K., Begotti, R., Sanches, I., Carvalho, A. X. Y., Coutinho, A., Esquerdo, J., Antunes, J., and Arvor, D. (2019). Land cover change maps for Mato Grosso state in Brazil: 2001–2017 (version 3). DOI: 10.1594/PANGAEA.899706.

[2] Hijmans, R. J. et al. (2020). ***raster**: geographic data analysis and modeling*, https://CRAN.R-project.org/package=raster. R package version 3.4-5.

[3] IBGE (2019). Sistema IBGE de recuperação automática. https://sidra.ibge.gov.br/. July 24, 2019.

[4] Pebesma, E. (2018). Simple features for R: standardized support for spatial vector data. *The R Journal*, 10(1):439–446, DOI: 10.32614/RJ-2018-009.

[5] R Core Team (2021). *R: a language and environment for statistical computing*. R Foundation for Statistical Computing, Vienna, Austria, https://www.R-project.org/. Version 4.1.1.

[6] Simoes, R., Picoli, M. C. A., Câmara, G., Maciel, A., Santos, L., Andrade, P. R., Sánchez, A., Ferreira, K., and Carvalho, A. (2020). Land use and cover maps for Mato Grosso state in Brazil from 2001 to 2017. *Scientific Data*, 7(1):1–10, DOI: 10.1038/s41597-020-0371-4.

[7] Tennekes, M. (2018). tmap: thematic maps in R. *Journal of Statistical Software*, 84(6):1–39, DOI: 10.18637/jss.v084.i06.

[8] Vicente-Serrano, S. M., Beguería, S., and López-Moreno, J. I. (2010). A multiscalar drought index sensitive to global warming: the Standardized Precipitation Evapotranspiration Index. *Journal of Climate*, 23(7):1696–1718, DOI: 10.1175/2009jcli2909.1.
